# The role of regulatory T cells and follicular T helper cells in HBV infection

**DOI:** 10.3389/fimmu.2023.1169601

**Published:** 2023-05-19

**Authors:** Nengqi Lin, Wei Yin, Heather Miller, Maria G. Byazrova, Andrés A. Herrada, Kamel Benlagha, Pamela Lee, Fei Guan, Jiahui Lei, Quan Gong, Youqing Yan, Alexander Filatov, Chaohong Liu

**Affiliations:** ^1^ Department of Pathogen Biology, School of Basic Medicine, Tongji Medical College and State Key Laboratory for Diagnosis and Treatment of Severe Zoonotic Infectious Disease, Huazhong University of Science and Technology, Wuhan, China; ^2^ Wuhan Children’s Hospital, Tongji Medical College, Huazhong University of Science and Technology, Wuhan, China; ^3^ Department of Research and Development, BD Biosciences, San Jose, CA, United States; ^4^ Laboratory of Immunochemistry, National Research Center Institute of Immunology, Federal Medical Biological Agency of Russia, Moscow, Russia; ^5^ Lymphatic Vasculature and Inflammation Research Laboratory, Facultad de Ciencias de la Salud, Instituto de Ciencias Biomédicas, Universidad Autónoma de Chile, Talca, Chile; ^6^ Université de Paris, Institut de Recherche Saint-Louis, EMiLy, Paris, France; ^7^ Department of Paediatrics and Adolescent Medicine, Li Ka Shing Faculty of Medicine, The University of Hong Kong, Hong Kong, Hong Kong SAR, China; ^8^ Department of Immunology, School of Medicine, Yangtze University, Jingzhou, China; ^9^ Clinical Molecular Immunology Center, School of Medicine, Yangtze University, Jingzhou, China; ^10^ Department of Infectious Disease, Wuhan No.7 Hospital, Wuhan, China

**Keywords:** HBV, regulatory T cell, follicular T helper cell, infection, potential therapy

## Abstract

Hepatitis B has become one of the major global health threats, especially in developing countries and regions. Hepatitis B virus infection greatly increases the risk for liver diseases such as cirrhosis and cancer. However, treatment for hepatitis B is limited when considering the huge base of infected people. The immune response against hepatitis B is mediated mainly by CD8+ T cells, which are key to fighting invading viruses, while regulatory T cells prevent overreaction of the immune response process. Additionally, follicular T helper cells play a key role in B-cell activation, proliferation, differentiation, and formation of germinal centers. The pathogenic process of hepatitis B virus is generally the result of a disorder or dysfunction of the immune system. Therefore, we present in this review the critical functions and related biological processes of regulatory T cells and follicular T helper cells during HBV infection.

## 1 Introduction

Hepatitis B, resulting from hepatitis B virus (HBV) infection, is one of the leading health challenges worldwide (1). Globally, it is estimated that 248 million people are chronically infected out of a total of about 2 billion people with hepatitis B, which means that about 20% of them will die from cirrhosis or secondary liver cancer (2). It is noteworthy that hepatitis B transmission in developing countries has shown moderate or high prevalence levels in the past few years (3), revealing a need to enhance our concern about this vital health issue.

HBV is a DNA virus that has an envelope with receptors specific for infecting hepatocytes (4). In 1965, Blumberg et al. first identified HBV antigens in Australian Aborigines by antigen testing and, thus, named them the “Australian Antigen” (5). In 1970, viral particles were first observed by electron microscopy by Dane et al. and three types of HBV particles were detected in patient sera (6).

The immune system is crucial during HBV infection. The pathogenesis of the associated liver disease depends on the dynamic balance between viral replication versus the host’s immune response (7). T lymphocytes are the most critical and dominant immune cells involved in fighting viral infections. However, the cytotoxic functions of CD8+T cells that eliminate HBV is also associated with hepatocyte damage (8). Additionally, CD4+ T cells activate and maintain CD8+ T cell responses and promote HBV-specific humoral immune responses (9).

Regulatory T (Treg) cells are capable of inhibiting excessive immune responses induced by effector T cells, including CD8+ and CD4+ T cells (10). Several studies have shown that Treg cells proliferate dramatically during chronic HBV infection and suppress the anti-HBV immune response (11, 12). It is assumed that Treg induction is initiated by HBV infected hepatic stellate cells (HSC) that produce TGF-β, which promotes the differentiation of Treg cells (13).

Follicular T helper (Tfh) cells are a subpopulation of CD4+ T lymphocytes that regulates the adaptive humoral immunity by promoting B lymphocytes to produce antibodies (14). A number of studies have shown that patients with chronic HBV infection have specific antiviral B cell defects (15–17). Furthermore, follicular T helper cells have the potential to promote plasma cytogenesis as well as HBs antigen-specific B-cell responses (17).

This review focuses on the series of immune processes activated by HBV invasion of hepatocytes, giving a detailed look at the mechanisms of Treg and Tfh cells. Additionally, we also describe the potential treatment options for hepatitis B based on these physiological mechanisms.

## 2 HBV infection

One HBV particle consists of an envelope of three related surface proteins and lipids plus an icosahedral nucleocapsid of approximately 30 nm in diameter (18). An HBV virus typically encodes several specific proteins that regulate biological processes within the infected cell, ensuring its ability to proliferate. The HBV genome formed by incomplete double-stranded circular DNA contains four overlapping open reading frames (ORF) that make up the genes- S, C, P, and X (19). The S gene encodes the viral envelope protein (surface antigen protein, HBsAgs) while the C gene encodes the core protein (21 k-Da) and pre-core (25 k-Da) protein (HBcAgs) (20). Viral DNA polymerase is a reverse transcriptase and is encoded by the P gene (HBpAgs) (21). The X gene encodes the most vital X protein (HBxAgs) that is involved in cell signaling, transcription and calcium signaling pathways, which affects a range of cellular activities such as apoptosis or proliferation (22).

HBx has been reported to promote viral replication by regulating the proliferation process of hepatocytes (22–25). Gearhart et al. first showed this in rat hepatocytes, whereby HBx was capable of inducing normal hepatocytes to reach the G1 phase of the cell cycle (22). There are evidence that HBx mediates hepatocarcinogenesis by affecting cellular signaling pathways. Accordingly, Chin et al. showed this by delivering a replication-competent HBV system into Huh7 and primary marmoset hepatocytes utilizing a recombinant adenovirus system, which activated MAPK/Akt pathways that resulted in uncontrolled cell cycle progression (26). Moreover, Kim et al. first reported that HBx promotes actin polymerization to enhance metastasis of hepatocellular carcinoma cells by interacting with calmodulin (CaM) to regulate the level of cofilin, an actin depolymerizing factor (27). In tumor cells from patients suffering HBV-induced liver cancer, Wang et al. detected upregulated levels of Sirtuin1, a crucial regulator of various signaling pathways (28). More studies have also pointed out that HBx, acting as a trans-activator, can also activate signaling pathways such as NFAT, CREB/ATF, Wnt/β-catenin and nuclear factor-κB (29–33).

## 3 Immunopathogenesis by HBV infection

### 3.1 Antigen-driven humoral immunity

Despite the general consensus that in HBV infection the T-cell immune response predominates, a growing number of studies are increasingly recognizing the role of B cells and antibody responses. Overall, B cells are not directly involved in virus clearance, but rather secreted antibodies bind to specific HBV proteins and mark the virus for removal (34).

There are two critical viral antigens. One is a 21 kDa protein which forms a dimer and assembles into a multimeric shell called the core-antigen (HBcAg), while the other is a 17 kDa protein called the e-antigen (HBeAg), which also forms a dimer but does not assemble into a structure (35). Both are variants of the same protein presenting two clinically essential and non-cross-reactive antigens, with HBcAg emerging in the pre-infection period in the form of particles that assemble the capsid as well as HBeAg in the non-particulate form (36). Currently, HBsAg is utilized as a protective antigen in vaccines, while the role of HBcAg and HBeAg is mainly diagnostic and to assess the course of the disease (37).

Deficiency in specific B-cells has been reported during chronic hepatitis B infection (15), affecting HBsAg-specific and HBcAg-specific B cells differently. Interestingly, Le Burt et al. utilized a fluorescent dye coupled to HBcAg for direct detection of isolated HBcAg-specific B cells from HB patients. This approach revealed that the HBcAg-specific B cells appeared more frequently than the HBsAg-specific, and were capable of secreting antibodies more potently *in vitro* (38). Corresponding to this defect in HBsAg-specific Ab secretion is suppressed activation of memory B cells (atMBC). Burton et al. found that chronic HB infection is enriched in atMBC and expresses various inhibitory receptors like PD-1 and FcRL5, thereby reducing the potential for differentiation into plasma cells (15).

In addition, the initiation of humoral immune responses in the liver is also closely linked to IL-21 secretion by Tfh cells, which will be explained further in subsequent sections.

### 3.2 T cell depletion

T cells serve a vital role in the immune clearance of antiviral responses, participating and coordinating multiple aspects of the body’s adaptive immunity. T cells mature from bone marrow progenitors that subsequently migrate to the thymus where they are negatively or positively selected for reactivity to specific markers prior to being exported to the periphery. Immature T cell populations in the thymus are classified by surface CD4 and CD8 receptors in the following stages: double negative (CD4-CD8-), double positive (CD4+CD8+) and single positive (CD4+CD8- or CD4-CD8+) (39). After binding selectively with the self-MHC and less intensely to self-peptides, the cells avoid programmed death and emerge as naïve CD8+ or CD4+ T cells (40). Peripheral T cells are comprised of several subsets, including naïve T cells stimulated by new antigens and memory T cells activated from previous antigens, as well as Treg cells that control excessive autoimmunity (41).

CD4+ T lymphocytes typically undergo differentiation into various subpopulations to aid in virus elimination upon infection. Naïve CD4+ T cells specifically identify complexes of MHC class II molecules and antigens presented by activated antigen-presenting cells (APC). In particular, activated APCs triggered by pattern recognition receptors (PRR) upregulate MHC II, co-stimulatory molecules (e.g.CD80, CD86) and pro-inflammatory factors (e.g. IFN I, TNF, IL-1, IL-6 and IL-12), which is followed by migration to the local lymph nodes for interaction and activation of virus-specific CD4+ T cells (42, 43). Antiviral CD4+ T cells mainly present with a Th1-phenotype and secrete large amounts of IFN-γ, TNF-α, IL-2 and express T-bet under the induction of IL-12 and IFN-γ. The antiviral effect of IFN-γ and TNF-α is based on phagocytosis of infected cells by macrophages, which are destroyed in endosomal compartments by nitric oxide (43–45). In addition, Th2 (IL-4 releasing) cells assist humoral immunity and suppress Th1 responses, while Th17 cells (IL-17 releasing) drive inflammatory responses to recruit neutrophils during viral infection (46–48). Further, CD4+ T cells also aid and sustain the functions of CD8+ T cells, possibly through CD40L-CD40 interactions that activate APCs (e.g. type 1 dendritic cells) including cytokines-release (e.g.IL-12 and IL-15) and co-stimulation, which drives the differentiation of CD8+ T cells to CTLs (49–52). Chronic HBV infection leads to depletion of Th1 cells, which is possibly attributed to the observed CD4+ T cell exhaustion. Also, several studies point to reduced secretion of Th1-related cytokines by CD4+ T cells as potentially contributing to Th1 cell depletion (45, 53–55). Additionally, some studies suggest that the upregulation of the Th2 cell ratio may further enhance Th1 cell depletion (45, 56–58), as HBeAg induces Th2 cell generation. CD4+ Treg cells drive T-cell depletion, which is also significant and will be discussed later.

It has been demonstrated that CD8+ T cells are the main effector cells associated with eliminating viruses and causing disease. The viral elimination is achieved through cytolysis of infected hepatocytes and a non-cytolytic process that involves the secretion IFN-γ and TNF- α (59). Robert et al. found dramatic depletion of CD8+ T cells, while essentially no change in CD4+ T cells during acute HBV infection in chimpanzees (60). Cell depletion in chronic viral infections is associated with high expression of co-suppressor molecules (61–63), such as CTLA-4/CD80(or CD-86) and PD-1/PD-1L. The high expression of co-inhibitory molecules in T cell exhaustion, such as PD-1, may cause T cell depletion by inhibiting glycolysis and glutamine activity while reducing the expression of mTOR-mediated GLUT1 and glutamine transporter proteins, thus promoting cellular reprogramming to shift fuel from glucose to fatty acids, which results in excessive accumulation of ROS due to increased β-oxidation, thereby damaging mitochondria and invoking apoptosis (64–69). Yan et al. recently identified a significant contribution by CCL19 in revitalizing CD8+ T cells in HBV infection. They found that CCL19-mediated interactions between CCR7 and PI3K/Akt promote cell proliferation (70). Kathrin et al. have currently proposed that the HBV-specific CTL marker, Thymocyte Selection-Associated High Mobility Group Box (TOX), can identify distinct subsets during chronic infection, such as higher TOX expression in HBVcore18-specific T cells and lower TOX expression in HBVpol455-specific and exhausted T cells (71).

Major effects and mechanisms of various T-cell subsets during HBV infection are shown in **Table 1**.

**Table 1:** General changes and mechanisms major T-cell subsets during HBV infection.

T-cell Subset	HBV-Infection stage	Change	Mechanism
Th1	Acute	↑	CD4+ T cells recognize MHC II molecular complexes and APC-presented antigens and are then activated and secrete large amounts of IFN-γ, TNF-α and IL-2 (42, 43).
	Chronic	↓	1.Decreased secretion of Th1-related cytokines (45, 53–55); 2.HBeAg induces Th2 cell formation and thus indirectly suppresses Th1 formation (45, 53–55); 3. Inhibition due to Treg overproliferation (based on PD1 signaling, CTLA4 signaling and cell contact, etc.) (72)
Th2	Acute	↑	Th2 releases IL-4, which assists humoral immunity and suppresses Th1 responses (46–48).
	Chronic	↑(Th2/Th1↑)	HBeAg induces Th2 cell formation; (45, 56–58)
Th17	Acute	Th17/Treg↑	Th17 secretes IL-17/22/23 to recruit neutrophils to promote inflammation (73–75).
	Chronic	Th17/Treg↓	Significant proliferative and anti-inflammatory effects of Tregs.
Treg	Acute	↓	Inhibition by pro-inflammatory factors
		↑	Inflammatory transformation (IL-17A+ Foxp3+ Treg↑)
	Chronic	↑	Shown in **Table 2**
Tfh	Acute	↑	To drive anti-virus humoral immunity
	Chronic	↓	Shown in **Table 3**
CD8+T	Acute	↑	CD4+ T cells drive CTLs activation which are the main effector cells for virus elimination
	Chronic	↓	High expression of co-repressed molecules (i.e.PD-1/PD-1L and CTLA-1/CD80 or CD86) (61–63)

The symbol "↑" indicates active differentiation of this cell subset or an increase in the ratio between subsets, while "↓" indicates inhibited differentiation of this cell subsets or a decrease in the ratio between subsets.

## 4 Regulatory T Cell in hbv infection

### 4.1 Origin and classification

In general, Treg cells refer to phenotype-specific CD4+CD25+Foxp3+ T cells, which play key roles in immune tolerance and autoimmune diseases, as well as tumorigenesis. Treg cells have two origins; natural regulatory T cells (nTreg), that develop directly from thymocytes and inducible regulatory T cells (iTreg), derived from peripheral mature T cells. It has been found that the formation process of nTregs can be divided into two steps (76). Initially, CD4 single-positive T cells upregulate the expression of IL-2 receptor (i.e.CD25) and TNF receptors (i.e. GITR, OX40 and TNFR2) and with TCR stimulation, differentiate into CD25+Foxp3- Treg progenitor cells. Next, upregulation of Foxp3 expression leads to the transformation of progenitor cells into mature nTreg cells (77). The study by Rafal et al. focusing on Foxp3+CD4+CD25+ thymocytes versus CD4+ naïve T cells in TCR-transgenic mice, demonstrated a more significant TCR diversity in the former, indicating that more diverse TCRs on Treg can match the specificity of naive CD4+ T cells to self and foreign antigens, contributing to the regulatory role of Treg (78). Foxp3+ iTreg cells differentiate under more diverse conditions, possibly in the lamina propria of the gut in response to microbiota and food antigens, as well as in chronically inflamed tissues, or in tumors and transplanted allogeneic organs (79–83). For iTreg differentiation, it was demonstrated in mice and humans, that naïve and memory T cells can transform into iTreg cells under TGF-β stimulation (83–85). In summary, nTreg cells prevent or regulate excessive autoimmunity, while iTreg cells primarily suppress the immune response to external antigenic stimuli or inflammatory autoimmune reactions.

### 4.2 Molecular mechanism

Treg cells have numerous surface marker molecules, including CD25, CD62L, CD103, CTLA-4, and GITR. CD25 is a characteristic surface molecule of Treg cells and has a significantly high expression in nTreg cells (86). Interleukin 2 (IL-2) was first considered to be an integral T-cell activator, yet, researchers found that mice lacking IL-2 or its receptor gene exhibited severe autoimmune symptoms instead of the expected immunodeficiency (87, 88). Subsequently, Sharfe et al. found that patients with mutations in the IL-2 receptor alpha chain (i.e. CD25) had invading lymphatic tissue extensively in the lung, liver, gut, and bone, exhibiting an uncontrolled autoimmune attack (89). IL-2 signaling has been found to have a direct effect on Treg cells. The IL-2 receptor (IL-2R) has a low-affinity dimeric form consisting of CD122(IL-2Rβ) and cytokine receptor gamma chains (γc or CD132) as well as a high-affinity trimeric form with the addition of CD25 (IL-2Rα) to the dimeric form (90). CD25 in the trimer is not directly involved in signaling, but rather boosts the affinity of the IL-2R for the ligand by 10-100 fold (90). For binding to the trimeric form, IL-2 first interacts with CD25, which induces a conformational change in IL-2 that greatly increases its binding affinity to CD122 and γc, then following the binding of IL-2 to the trimeric form of IL-2R, the signaling motifs in the cytoplasmic tail of CD122 and γc mediate signaling pathways such as JAK-STAT, PI3K-AKT and MAPK (87). Sophie et al. suggested an endogenous mechanism for regulating IL-2 signaling, whereby Arg35 of CD25 at the IL-2 binding site is catalyzed by ARTC2.2 for ADP-ribosylation. In an inflammatory environment, damaged cells release NAD+, which promotes ADP-ribosylation of CD25, so that IL-2 tends to bind to the low-affinity receptor CD122/132 on NK cells and CD8+ T cells to drive cell proliferation. In contrast, in a non-inflammatory environment, IL-2 tends to bind to high-affinity receptor CD25 on Tregs, thereby depleting IL-2 and exerting immunomodulatory effects (91).

It has also been suggested that the suppressive effect of nTreg on CD4+ T cells or CD8+ T cells *in vitro* is dependent on cell contact, not cytokines (92). CTLA-4 is a co-repressor molecule that mediates the regulatory mechanism in Treg cells. Indeed, CTLA-4-deficient mice and humans both have intense autoimmunity (93–96). Several potential regulatory processes exist for the inhibition of T cells by CTLA-4, with a number of mechanisms based on the interaction between CTLA-4 on Tregs with CD80/CD86 on antigen-presenting cells (APCs). One possibility is that Treg-expressed CTLA-4 depletes CD80/CD86 on APCs *via* inducing endocytosis. This competitive depletion of ligands prevents CD28 co-stimulation with effector T cells, thereby inhibiting T-cell activation (97–100). This hypothesis is based on the fact that CTLA-4, unlike the surface receptor CD28, is highly endocytic and occurs predominantly in intracellular vesicles, while undergoing little conformational change upon binding to ligands (101). Tai et al. utilized confocal microscopy to examine the intracellular localization of the transgenic CTLA-4 protein in conventional T cells and Tregs, and found abundant Golgi retention in conventional T cells, which was probably due to the lack of high-affinity TCR interactions, while Tregs showed intravesicular distribution close to the plasma membrane (102). It has also been suggested that the interaction of CTLA-4 with CD80/CD86 of APCs (DCs, for example) induces indoleamine 2,3-dioxygenase (IDO), which is responsible for tryptophan metabolism in APCs, in parallel with the inhibition of kynurenine production, thus promoting APC-apoptosis (103, 104). A more recent study showed that *in vitro* blockade of CD80 by soluble CTLA-4 increased free PD-L1 release on splenic DCs. This is based on the formation of cis-CD80/PD-L1 dimers on DCs, and the soluble CTLA-4 disrupts the original heterodimer by promoting CD80 homodimerization, ultimately releasing free PD-L1 and upregulating the intensity of PD-1 inhibition (72). In conclusion, the specific mechanism of CTLA-4 involved in Treg cell suppression needs to be further investigated and validated, although it has been shown that CD80/CD86 could be targets of CTLA-4.

It is essential to reduce Treg responses if an effective immune response is needed. Toll-like receptors (TLR) have been reported to modulate the inhibition of Tregs. A study by Li et al. demonstrated that TLR8 signaling inhibits normal metabolism in human Treg cells, including inhibition of key glycolytic enzymes and also inhibition of glucose uptake through downregulation of glucose transporter protein (GLUT) 1 and GLUT3 (105). Another possible mechanism is APC-mediated, with either lipopolysaccharide (LPS)-TLR4 binding or CpG dinucleotide-TLR9 binding, which activates APC production of IL-6 or GITR ligands, that in turn causes effector T cells to express IL-6 receptors and GITR, potentially making them insensitive to Tregs (106–108).

### 4.3 Role in HBV infection

Tregs cells suppress the antiviral T cell response to acute or chronic infections, potentially leading to viral persistence or prevention of autoimmunity (109). In chronic HBV infection, patients have a weak immune response to HBV, a fact that Jeroen et al. suggested could be attributed to an increased ratio of Tregs in the peripheral blood, resulting in diminished antiviral effect (110). Th17 cells are a subset of CD4+ T cells that secrete IL-17 and are derived from the same naïve cells as Treg cells, but secreting proinflammatory factors that are closely associated with inflammatory responses and antimicrobial immunity. The ratio of Th17/Treg cells has relevance in evaluation of the immune response process, as Th17 secretes pro-inflammatory factors (IL-17, IL-22 and IL-23) to recruit neutrophils to promote inflammation at the site of infection, while Treg cells produce anti-inflammatory factors (IL-10 and TGF-β) to suppress inflammation (73–75). Liang et al. found that the expression of Th17 effectors was significantly higher in acute HBV infection (AHB) than in chronic infection (CHB), with prominent proliferation of Treg cells observed in chronic infection (111). The possible reason for this contrasting cell ratio might be a variation in the differentiation microenvironment during HBV infection, with higher levels of IL-23 and IL-6 that promote Th17 cells in AHB than in CHB, as well as distinctly higher levels of TGF-β and IL-2 in CHB that sustain Treg cell differentiation (111). Feng et al. found that peripheral blood Tregs cells expressed IL5-Rα and PD-1 antigens during chronic infection, which suppressed peripheral blood mononuclear cells (PBMCs), leading to immune tolerance to chronic HBV infection (112). However, this cell ratio is not definitely true in HBV-induced acute hepatitis. In a more recent study, Le et al. examined the Treg/Th17 ratio in HBV patients with different progression (113). The results showed that the Treg/Th17 ratio was highest in severe hepatitis episodes but lowest in chronic HBV infection and healthy individuals, which is contradictory to previous studies. This might be attributed to the significantly higher IL17A+ Foxp3+ Treg subset detected in the severe hepatitis group, which suggests that Treg cells have dual and opposite functions in immunosuppression and inflammation enhancement during HBV infection. This non-traditional Treg cell secretes pro-inflammatory factors (e.g.IL-17A) under inflammatory conditions (e.g.IL-6/IL-23/IL-1β/TLR stimulation) that exacerbate severe damage during infection instead (114). The inflammatory transformation of Tregs has been reported in some pathological changes due to dysregulation of immune homeostasis, such as psoriatic arthritis, which may be an important factor in severe inflammatory damage in acute HBV infection (114, 115). Further, Treg cells in HBV infection can also inhibit the body’s anti-tumor immune response, thus promoting the progression of hepatitis B to hepatocellular carcinoma (116).

Currently, there is no conclusive evidence of how HBV viruses induce Treg responses. One potential mechanism is that hepatitis B virus replication induces soluble heat shock protein 60 (HSP60) production in infected hepatocytes to enhance the effect of Treg cells. Kondo et al. constructed an *in vitro* HBV replication system and detected expression of soluble HSP60 in HBV-replicating hepatocytes (117). Heat shock proteins (HSP) are constitutively expressed in all cells and involved in biological processes such as protein folding and mediating protein translocation across membranes. Several external conditions (e.g. heat shock, UV radiation, and bacterial or viral infection) induce the production of HSP, further engaging in a range of immune processes (118, 119). In particular, HSP60 has been reported to activate Treg function by inducing the production of TGF-β and IL-10 (120), as well as initiate the Toll/IL-1 signaling pathway (121). *In vitro* studies also found that HBeAg stimulated upregulation of TGF-β expression in mouse splenocytes, which led to differentiation into Treg cells (122). In addition, a new study suggested that the immune mechanism of Treg in HBV infection may be related to furin (123). As an endoprotease, furin is involved in production of some cellular functional proteins and plays a key role in virus infection (124). For example, it has been proven that the spike protein of SARS-CoV-2 is cleaved by furin to promote virus-cell fusion (125). Furin is engaged in the precursor processing of TGF-β1 and is also expressed abundantly in Treg. Qiu et al. found that furin expression and TGF-β secretion were significantly increased in the *in vitro* co-culture environment of Treg and HBV-infected hepatocytes, which indicated that furin and TGF-β1 could form a positive feedback loop to activate Tregs in HBV infection (123).

**Figure 1:**
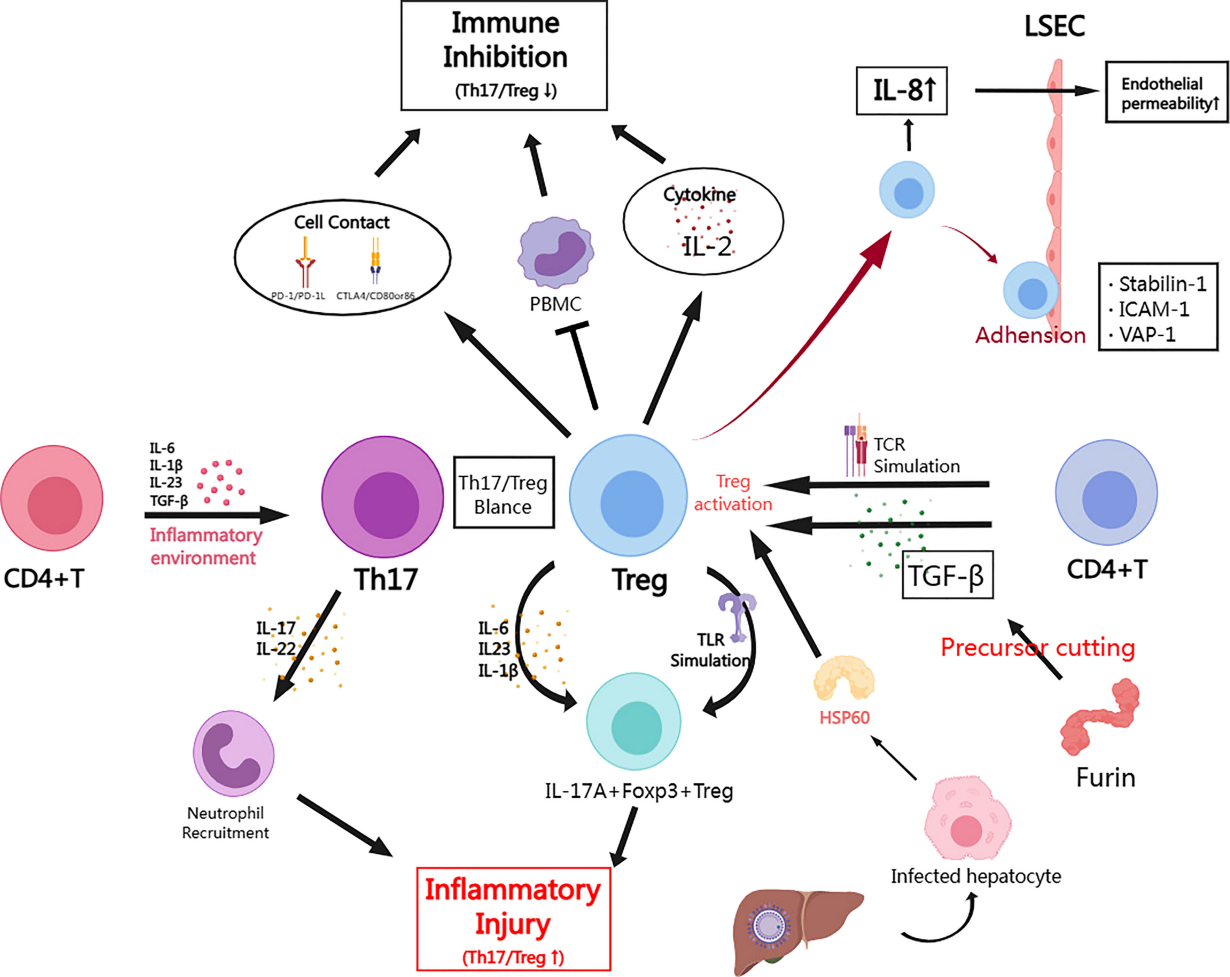
The role of Treg cells in HBV infection and the mechanism of immune regulation. (By MedPeer) In the inflammatory microenvironment caused by acute HBV infection, CD4+ T cells are activated by pro-inflammatory factors (IL-4/16/23, TGF-β, etc.) and differentiate into Th17. While in chronic infection, CD4+ T cells can differentiate or be induced to form Treg in response to TCR signal or TGF-β stimulation; Tregs inhibit the immune response through cell contact mediated by PD-1 or CTLA4 signal, or through secreting inhibitory cytokines (e.g. IL-2). Tregs may also suppress peripheral blood mononuclear cells (PBMCs) to exert indirect suppressive effects; Th17 mainly secretes pro-inflammatory factors (IL-17/22) to recruit neutrophils causing inflammatory injury; Tregs can also undergo inflammatory transformation towards IL-17A+ Foxp3+ Tregs in HBV infection, which can be driven by cytokines (IL-6/23/1β) or Toll-like receptor (TLR) pathway; Furin is highly expressed in Treg and is also involved in the cleavage of TGF-β precursors, which has a significant positive cycling relationship with TGF-β in HBV infection models; In acute hepatitis injury, infected hepatocytes secrete HSP60 to promote Treg activation; HBV induces Tregs to increase IL-8 secretion to improve endothelial permeability thereby promoting Treg migration and infiltration; Stabilin-1, ICAM-1 and VAP-1 mediate adhesion between Tregs and LSECs, which facilitates Treg migration.

One view on how hepatitis B virus induces Treg accumulation in the liver is that HBV infection can activate IL-8 expression. The study by Zhang et al. revealed that HBV induces IL-8 expression through the MEK-ERK pathway which is mediated by HBx (126). It was also found that the IL-8/CXCR1 axis could reduce intercellular tight junctions thereby increasing endothelial permeability, which resulted in significant infiltration of Tregs in the hepatic microenvironment (126). Previous studies has reported that liver sinusoidal endothelial cells (LSECs) can induce Tregs generation either by secreting TGF-β or by anchoring exogenous LAP/TGF-β to the cell membrane *via* GARP (127). Zhang et al. further found that CXCR1 could interact with GARP and increase the GARP expression, which indicated that LSECs could induce intrahepatic Tregs *via* the IL-8/CXCR1/GARP pathway (126). In conclusion, the HBx-activated IL-8/CXCR1 axis may significantly influence the formation of the immune microenvironment in HBV infection, with important implications for the progression of HBV infection and even HBV-associated hepatocellular carcinoma. Regarding the recruitment process of Treg cells into the human liver, it is assumed that stabilin-1 mediates the migration of T cells through the LSECs (128). Stabilin-1 is a multifunctional scavenger receptor expressed in human spleen sinusoidal endothelial cells, as well as in macrophages and blood sinusoidal endothelial cells (129). Shetty et al. first reported the expression of stabilin-1 in inflammatory sites of LSECs, which participate in transendothelial and transcytotic movement of Tregs. Interestingly, the upregulation of stabilin-1 levels was found to be independent of pro-inflammatory cytokines, such as IL-4, but rather influenced by hepatocyte growth factor (HGF), indicating that this may occur as a result of tissue remodeling in the course of inflammation or cancer rather than in response to pro-inflammatory signals (128). In addition, transendothelial migration of Treg cells involves the two adhesion factors, ICAM-1 and VAP-1 (130), with ICAM-1 mediating the adhesion of Tregs in the LSEC (131). These observations were also confirmed in a study by Shetty et al. (128) (**Figure 1**)

Effects and mechanisms of Tfhs in HBV infection are shown in **Table 2**.

**Table 2:** Effects and mechanisms of Tregs in HBV infection.

Study	Related Protein	Mechanism
Liang, 2012 (111)	IL-1β、IL-23、IL-6、TGF-β1、IL-2	Treg/Th17 imbalance is generally seen in chronic infections, while Treg increase causes immunosuppression and persistent viral infections. Th17 increase is seen in acute infections and predisposes to inflammatory damage.
Feng, 2007 (112)	IL-5Rα、PD-1	Suppression of peripheral monocytes, leading to immune tolerance to chronic HBV infection
Le, 2023 (113)	IL-17A (IL-17A+ Foxp3+ Treg↑)	1. Pro-inflammatory factor induction (IL-6, IL-23 and IL-1β) (114); 2. Toll-like receptor (TLR) stimulation (114); 3. Resulting in Inflammatory injury;
Kondo, 2010 (117)	sHSP60	1.Induction of TGF-β and IL-10 (120); 2.Activation of Toll/IL-1 signaling pathway (121);
Tang, 2020 (122)	HBeAg	HBeAg induces TGF-β1 expression.
Qiu, 2022 (123)	Furin	Furin is involved in TGF-β generation;
Zhang, 2021 (126)	IL-8/CXCR1 axis	1.HBV induces IL-8 expression mediated by HBx *via* MEK/ERK pathway; 2.IL-8/CXCR1 axis increases endothelial permeability; 3.Intrahepatic induced Tregs formation *via* IL-8/CXCR1/GARP pathway;
Shetty, 2011 (128)	stabilin-1	Stabilin-1 mediates the migration of Treg across the endothelium and across cells.
Lalor, 2002 (130)	ICAM-1、VAP-1	Involvement of adhesion during transendothelial migration

## 5 Follicular T helper cell in HBV infection

### 5.1 Origin and molecular mechanisms

Tfh cells are a subset of CD4+ T cells existing in the follicles of peripheral lymphoid organs and are critical for the immune response of B cells. Tfh differentiation originates from the interaction between naïve CD4+ T cells and myeloid APCs (132). The Tfh differentiation process involves multiple factors that can be divided into three stages. Early differentiation is regulated by IL-6, ICOS, or IL-2 stimulation, which mediates CXCR5, Bcl6 and other target protein expression. In particular, IL-6 regulates the expression of the transcription factor Bcl6, which promotes Tfh differentiation while inhibiting the transition to Th1, Th2 and Th17 (133). Another important cytokine that modulates the initial Tfh differentiation is IL-2, which induces STAT5 expression that inhibits Bcl6 expression and thus suppresses Tfh differentiation (134, 135). In addition, miR-19~72, a microRNA cluster, was reported to inhibit PI(3)K-inactivating phosphatases (PHLPP2 and PTEN), which are inhibitors of ICOS signaling (132, 136). The second stage of differentiation involves T cells interacting with antigen-specific B cells in the follicle, interfollicular zone, or follicular T-B boundary. At this point, Tfh and B cells are co-localized and have tight interaction as a result of the co-expression of CXCR5 (132, 137). The third stage of differentiation occurs in the germinal center (GC) and is characterized by having high expression of CXCR5, low expression of CCR7, elevated expression of CXCR4, low levels of SIP1R and low levels of PSGL1 on Tfh cells (132). After interacting with B cells, Tfh cells in the GC translocate to the next germinal center, remain in the follicles of adjacent B cells waiting to re-enter the same GC, or leave the GC to become memory Tfh cells (138).

Tfh cells drive the survival and differentiation of B cells in germinal centers (139). Tfh cells highly express CD40L, which by binding to the CD40 on B cells, activating signaling pathways to prevent apoptosis. Additionally, IL-21 and IL-4 produced by Tfh cells induce the proliferation and differentiation of GC B cells, with IL-21 upregulating Bcl6 expression in B cells to maintain survival, antibody maturation and differentiation towards plasma cells, and with IL-4 enhancing the intensity of glucose metabolism to sustain energy supply (139). A further essential contribution of Tfh cells to B cells is chemotaxis *via* high expression of CXCL14, the ligand for CXCR5, which recruits B cells for activation (140). Furthermore, Tfh cells also express SLAM-associated proteins (SAP), essential for antigen-specific T-B adhesion and signaling. The role of SAP has been reported to be independent of CD40-CD40L and CXCL14-CXCR5 interactions (141, 142). As an intracellular adaptor protein that regulates immune responses, SAP binds to members of the SLAM family of receptors, of which those expressed by CD4+ T cells and most significantly upregulated in Tfh cells include SLAM, Ly9, CD84 and Ly108 (142). Recent studies have revealed multiple roles of SAP in the interactions between Tfh cells and B cells. Foremost, SAP mediates T-B cell adhesion by binding to the cytoplasmic tail of SLAM family receptors and recruiting molecules such as the Src family kinase Fyn to mediate positive signaling (139, 143, 144). Secondly, SAP was also found to mediate the regulation of TCR signaling, for instance, positive signaling of SAP acting with Ly108 maintains ERK activation to amplify TCR signaling (139, 143). Moreover, SAP is also involved in the regulation of cytokine secretion, with SLAM shown to induce IL-4 production by Tfh cells (145). Inside the germinal center, B cells located in the light zone (LZ) upregulate SLAM expression while Tfh cells in the dark zone (DZ) overexpress SAP, resulting in elevated IL-4 production (139).

### 5.2 Role in HBV infection

Significant expansion of Tfh cells has been reported in HBV-infected patients (146–148). In addition, Simpson et al. found a high rate of peripheral blood Tfh cells in patients with systemic lupus erythematosus (SLE) and dry syndrome (SS), indicating that the expansion of Tfh cells may be a feature of impaired immunosuppressive function (149), while also reflecting a positive trend to humoral immunity, due to the important role of Tfh cells on antigen-specific B cells. The other powerful evidence is that Tfh cells export was tracked in healthy individuals after hepatitis B vaccination and found to be significantly elevated (150, 151). These suggest that circulating Tfh (cTfh) cells are involved in changes of humoral immunity during HBV infection. IL-33, as a member of the IL-1 cytokine superfamily, has been shown to drive the expression of Th2-related cytokines, which in turn drive humoral immune processes (152). Jiang et al. found that IL-33 treatment could activate CD4 + CXCR5 + Tfh to restore humoral immunity in HBV-infected mice. The mechanism may be that IL-33 binds to its receptor and induces the activation of STAT4, which enhances Bcl6 expression and thus promotes Tfh differentiation (153). So, are there more cytokines involved in the activation of Tfhs? Ayithan et al. showed that activation of toll-like receptor 8 could drive monocytes to secrete IL-12, which in turn promotes the differentiation of CD4+ T cells to Tfhs in the peripheral blood of HBV patients. Correspondingly, they found higher HBsAg concentrations in patients treated with TLR8 agonists, suggesting a new therapy (17). Interestingly, a similar mechanism was also found in the TLR7-mediated signaling pathway. Mori et al. found that TLR7 stimulator (GS-986) treated peripheral dendritic cells expressed OX40L and produced IL-6/IL-12, leading to the induction of Tfh cells from naïve CD4+ T cells (154).

Seroconversion is an important indicator of the course of chronic HBV disease. Seroconversion of HBeAg indicates suppression of viral replication, while seroconversion of HBsAg suggests functional cure. As an essential component involved in serological conversion, the change in the effect of Tfhs during HBV infection is of great significance. It has been reported that HBsAg-specific follicular helper T cells are distinctly defective in HBV-infected mice, which directly leads to a lack of HBsAb (155, 156). Based on the fact that this suppression of Tfh is likely due to Tregs inhibitory function, a therapeutic strategy was proposed in the study by Wang et al. (155) They utilized the humanized anti-CTLA4 monoclonal antibody ipilimumabz to block the inhibitory effect of Treg and found that the defective of HBsAg-specific Tfhs was improved and serum HBsAb concentration was raised. Tfh cells during chronic HBV infection may also have an essential impact on the seroconversion of HBeAg (157, 158). Vyas et al. found that when seroconversion occurred spontaneously in HBV patients, the incidence of seroconversion was higher for HBeAg than for HBsAg (158). Publicover et al. found that mice lacking IL-21 receptors on splenocytes failed to produce HBsAb, resulting in persistent HBV infection (159). Similar results were obtained in a study by Zhang et al. In addition, they proposed that upregulation of PD-1/PD-1L affected the seroconversion of HBeAg by providing inhibitory signals (157). Hu et al. found lower HBsAg in patients treated with PEG-IFN-α who had increased PD-1 hi CXCR5 + CD4 + T cells, suggesting that the increase in Tfh with high PD-1 expression promotes a decrease in HBsAg (160) It has also been suggested that PD-1 ^hi^ CD4 + CXCR5 + Tfh cells, may be involved in the pathogenesis of HBV-associated membranous nephropathy by promoting the secretion of corresponding antibodies leading to the deposition of antigen-antibody complexes (161). Complementarily, it has been reported that HBV may upregulate PD-1 expression in Tfh cells by promoting prostaglandin E2(PGE2) secretion in infected hepatocytes, while HBeAg and HBsAg secreted by HepG2.2.1.5, the cell line selected for the study, did not directly evoke variation in PD-1 levels (162). PGE2 is secreted by a variety of cells as well as acting on most components of the immune system to regulate the immune response or inflammatory response (163). In particular, as an upstream signaling molecule of PGE2, COX-2 has been reported to be upregulated by HBxAg to promote the proliferation of HepG2 cells (162, 164). In conclusion, CD4+ CXCR5+ T cells expand with increasing strength of humoral immunity at the beginning of HBV infection, whereas in patients with chronic hepatitis B infection, suppression of antigen-specific B cells may be associated with increased PD-1 expression in Tfh cells. In addition to PD-1/PD-1L, other signals may also affect the Tfh-B cell axis. Poonia et al. found that during chronic HBV infection, the overactivated Tfh population upregulates CD40L expression (16). In general, CD40/CD40L interaction is a key signal for Tfh to promote plasma cell formation and antibody secretion. However, excessive CD40L stimulation affects the direction of B cell differentiation, which inhibits terminal B cells differentiating into long-lived plasma cells (16).

**Figure 2:**
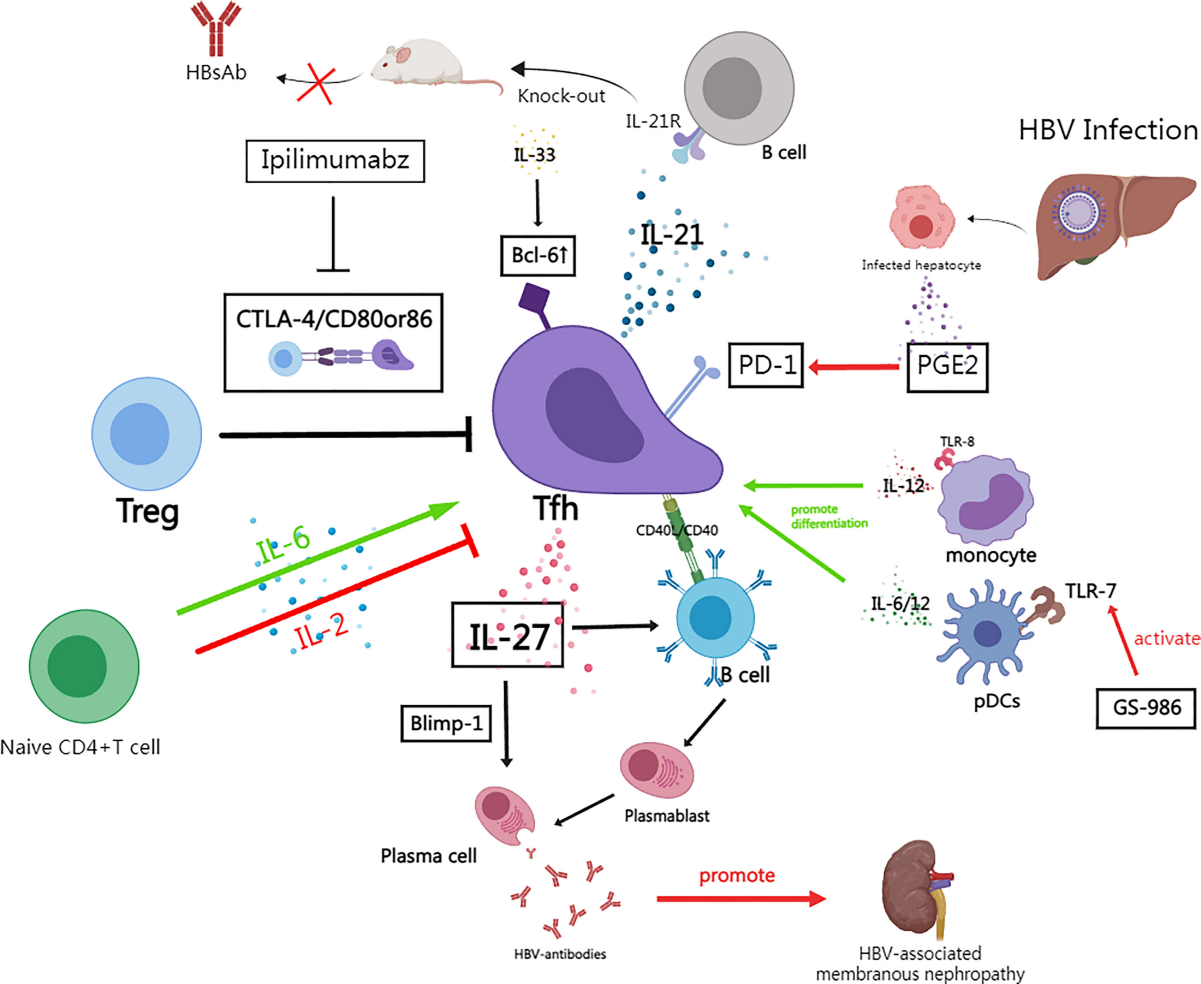
The role of follicular T helper cells in HBV infection and the mechanism of immune regulation. (By MedPeer). Naive CD4+ T cells are induced to differentiate into Bcl -6+Tfh in response to cytokines such as IL-6, while IL-2 is a suppressor of this process; In HBV infection, Tregs inhibit Tfh function through CTLA-4 signaling, and the human monoclonal antibody Ipilimumabz reverses this inhibitory effect by specifically blocking CTLA-4 signaling; Tfh secretes IL-21 to drive B cell responses while knockout IL-21R mice fail to secrete HBsAb; In HBV infection, increased secretion of PGE2 by damaged hepatocytes and other tissue cells promotes PD-1 expression of Tfh; In chronic HBV infection, Tfh can still secrete IL-27 to drive some humoral immunity despite the fact that IL-21+Tfh is significantly suppressed——IL-27 activates plasmablasts and plasma cells as well as activates Blimp-1 expression to maintain HBV-antibodies production; IL-33 enhances Bcl6 expression which promotes Tfh differentiation; Activation of TLR8 drives IL-12 secretion from monocytes which drives Tfh differentiation; TLR7 stimulator (GS-986) promotes pDCs to produced IL-6/12; Tfhs promote the formation of immune complexes and aggravates the progression of HBV-associated membranous nephritis.

A recent study also suggested that although Tfhs exhibit IL-21 deficiency but IL-27 secretion is not affected during HBV infection, which allows them to maintain a certain level of humoral immunity. Khanam et al. suggested that Tfhs induces formation of plasmablasts and plasma cells by secreting IL-27 (165). They also found that blocking the IL-27 pathway caused an extremely low expression of Blimp-1 (a protein which is important for antibody production), which indicates that IL-27 may be a potential promoter of Blimp-1. Interestingly, several studies have already reported the importance of detecting IL-27 expression in predicting the development of HBV infection (166–168). In summary, Tfhs during HBV infection maintain the secretion of HBsAg and total HBV-Ab by secreting IL-27 that interacts with B-cell surface homologous receptors to activate downstream signals, leading to the activation of plasmablasts and plasma cells and increased expression of Blimp-1 (165) (**Figure 2**).

Effects and mechanisms of Tfhs in HBV infection are shown in **Table 3**.

**Table 3:** Effects and mechanisms of Tfhs in HBV infection.

Study	Related Protein	Mechanism
Wang, 2018 (152)	CTLA4	Treg proliferation inhibits Tfh formation and blocking CTLA4 signal can reverse this inhibition.
Publicover, 2011 (156)	IL-21R	The mice lacking of IL-21R failed to produce HBsAb.
Hu, 2018 (160)	PD-1	Tfh with high PD-1 expression promotes a decrease in HBsAg.
Liu, 2014 (161)	antigen-antibody complexes	Tfh promotes the formation of immune complexes and aggravates the progression of HBV-associated membranous nephritis.
Sui, 2017 (157)	PGE2、PD-1	High expression of PGE2 of hepatocytes induces PD-1 expression of Tfhs.
Khanam, 2012 (160)	IL-27/blimp-1	1.Tfhs induces formation of plasmablasts and plasma cells by secreting IL-27; 2. IL-27 may be a potential promoter of Blimp-1.
Jiang, 2015 (153)	IL-33	1. IL-33 treatment could activate CD4 + CXCR5 + Tfh to restore humoral immunity in HBV-infected mice. 2. IL-33 might enhance Bcl6 expression which promotes Tfh differentiation.
Ayithan, 2021 (17)	TLR-8/IL-12	1. Activation of TLR8 drives IL-12 secretion from monocytes. 2. IL-12 drives Tfh differentiation in PBMC from HBV patients.
Mori, 2023 (154)	TLR-7、GS-986	TLR7 stimulator (GS-986) promotes pDCs to express OX40L and produced IL-6/IL-12.
Poonia, 2018 (16)	CD40L/CD40	Tfh overexpresses CD40L and leads to abnormal B-cell differentiation direction.

## 6 Potential treatment

Based on the above discussion, the process of reconstitution of the T-cell immune response is an overarching theme in the cure of hepatitis B. A recent study by Oliver’s team revealed the great potential of the TLR8 agonist GS-9668 for the treatment of HBV infection. *In vitro* treatment of peripheral blood mononuclear cells with GS-9688 resulted in a decrease in Treg cells frequency and an increase in CD4+ Tfh cells frequency (169). TLR serve an essential function in immune responses induced by some viral infections. In human respiratory syncytial virus infection, TLR8 is important for inducing TNF-α production that corresponds to recovery (170). Carolina et al. proposed a clinical treatment option through a combination of GS-4774 and tenofovir. The yeast vector-based GS-4774 vaccine contains hepatitis B virus S, X and C proteins. Both *in vivo* and *in vitro* experiments showed enhanced specific CD8+ T cell responses after vaccination with GS-4774 (171). Meanwhile, the yeast carrier component promotes the differentiation of naive T cells to Th17 cells, but not Treg cells, by inducing IL-1β, resulting in a decrease in Treg cell frequency (171, 172). In general, HBV treatment is mainly directed against viral proteins as well as infection-induced immune dysregulation. While various vaccines based on the former have been clinically validated, immunotherapy requires further clinical research.

## 7 Conclusion

Hepatitis B is receiving increasing attention as one of the major health threats worldwide. This review first described the HBV molecular biology and the mechanisms of hepatocyte invasion, and then introduced the immune mechanisms of the liver after hepatitis B virus infection, focusing on cellular immunity as well as humoral immunity. We further focused on the range of activities of Tregs and Tfh cells in HBV infection and suggested several potential mechanisms of HBV infection. However, there are still many questions that remain to be elucidated, such as how HBV infection actually regulates the proliferation of Treg cells and Tfh cells and their signaling pathways. Finally, immunotherapy targeting T cells may be a future direction for the treatment of hepatitis B, which requires further research on specific mechanisms and more data from clinical trials.

## Author contributions

NL, WY, HM, MB, AH, KB, PL, FG, JL are responsible for literature collection and manuscript writing. QG, YY, AF and CL, as the corresponding authors, provided ideas for this review and guided the writing. All authors contributed to the article and approved the submitted version.
